# Power Sharing, Capacity Building, and Evolving Roles in ELSI: The Center for the Ethics of Indigenous Genomic Research

**DOI:** 10.33596/coll.71

**Published:** 2020-11-18

**Authors:** Jessica Blanchard, Vanessa Hiratsuka, Julie A. Beans, Justin Lund, Bobby Saunkeah, Joseph Yracheta, R. Brian Woodbury, Erika Blacksher, Michael Peercy, Scott Ketchum, Christie Byars, Paul Spicer

**Affiliations:** 1 University of Oklahoma, US; 2 Southcentral Foundation, US; 3 Chickasaw Nation, US; 4 Missouri Breaks Industries Research, Inc, US; 5 University of Washington, US; 6 East Central University, US

**Keywords:** American Indian/Alaska Native, community engagement, community-based participatory research, ELSI, genomics

## Abstract

Persistent, unresolved issues stemming from a legacy of scientific exploitation and bio-colonialism have kept many tribal nations from participating in genomic research. The Center for the Ethics of Indigenous Genomic Research (CEIGR) aims to model meaningful community engagement that moves toward more inclusive and equitable research practices related to genomics. This article reflects on key successes and challenges behind CEIGR’s efforts to shape Ethical, Legal and Social Implications (ELSI) research in ways that are informed by Indigenous perspectives, to locate community partnerships at the center of genomics research, and to conduct normative and empirical research with Indigenous communities that is grounded in the concepts of reciprocity, transparency and cultural competency. The structure of CEIGR represents an important shift away from a traditional model centered on a university-based principal investigators toward a partner-centered research approach that emphasizes equity and community control by distributing power and decision-making across all CEIGR partner sites. We discuss three features of CEIGR that have contributed to this shift towards an equitable, community-driven partnership: 1) balancing local priorities with collective goals; 2) distributing power in ways that promote equitable partnerships; and 3) capacity building and co-learning across partner sites. The discussion of these three areas in this article speaks to a particular strength of our Center: the interdependence among partners and collective willingness to maintain a plasticity of leadership that creates space for all of our partners to lead, support, exchange and strengthen ELSI research.

Persistent, unresolved issues have kept many tribal nations from participating in genomic research (Hiratsuka et al., 2019). Genomic research is the study of the entire human genome, and includes functions of specific genes, genetic variation and disease risk, interactions between genes and the environment, and the development of new technologies to detect, diagnose and treat certain diseases. Apprehension among some tribal communities about participating in genomic research stems from a legacy of scientific exploitation and continuing bio-colonialism in Indigenous communities (Bowekaty & Davis, 2003; Christopher et al., 2011; Drabiak-Syed, 2010; Harding et al., 2012; Harry & Dukepoo, 1998; Hodge, 2012; Kelley et al., 2013; Morton et al., 2013; Strickland, 2006). The continued failure of researchers to engage Indigenous communities in ethical and inclusive ways (Claw et al., 2018; Hiratsuka et al., 2019; Popejoy & Fullerton, 2016) perpetuates mistrust and shapes ethical standards and perceptions of genetic research across some Indigenous communities today (Claw et al., 2018; Dillard et al., 2018).

Authentic American Indian/Alaska Native (AI/AN) community engagement is required to overcome generations of barriers and underrepresentation. The Center for the Ethics of Indigenous Genomic Research (CEIGR) aims to model meaningful community engagement in genomics research and move toward inclusive and equitable research practices. The specific research approaches and methods described in this article are specific to the Center’s research with AI/AN communities, but the lessons about partnerships and collaboration are applicable to all who endeavor more ethical and accountable community-engaged research. This article reflects on key successes and challenges behind the CEIGR’s efforts to shape Ethical, Legal and Social Implications (ELSI) research in ways that are informed by Indigenous perspectives, to locate community partnerships at the center of genomics research, and to conduct research with Indigenous communities that is grounded in the concepts of reciprocity, transparency, and cultural competency.

## Center for the Ethics of Indigenous Genomic Research- Reflections on the Growth of the Center

Center for the Ethics of Indigenous Genomic Research, a National Human Genome Research Institute-funded Center of Excellence in ELSI Research, emerged as a multidisciplinary research consortium focused on systematic inquiry into tribal concerns about genomic research. The Center is based on the collaborative working relationship between researchers at the University of Oklahoma (OU) and three research groups based in AI/AN communities led by Indigenous researchers: the Chickasaw Nation (CN); Missouri Breaks Industries Research Inc. (MB), an AI–owned private research organization; and, Southcentral Foundation (SCF), an AN tribal health organization based in Anchorage, Alaska (see Hiratsuka et al., 2019 for a full description of the Center’s formation and partners). This article describes how the attributes of persistence, flexibility, and shared leadership enabled CEIGR to embrace the challenges and dissimilarities among diverse members and work toward a strengths-based, partner-centered model for conducting collaborative research.

The structure of CEIGR represents an important shift away from a traditional model centered on a university-based PI toward a partner-centered research approach that emphasizes equity and community control by distributing power and decision-making across all CEIGR sites. This approach enhances collaborative partnerships and research capacity across diverse community sites, which in turn promotes research that is grounded in local community needs and concerns. Below, we discuss three features of CEIGR that have contributed to this shift towards an equitable, community-driven partnership: 1) balancing local priorities with collective goals; 2) distributing power in ways that promote equitable partnerships; and 3) capacity building and co-learning across partner sites.

## Balancing Local Priorities and Collective Goals

Since its inception, CEIGR has sought to develop research initiatives, some described below, that are inclusive of community-based investigators and prioritize community-driven initiatives (Woodbury et al., 2019a; Hiratsuka et al., 2020a; Reedy et al., 2020b). Establishing a partner-centered research agenda required identification of collective goals that could be implemented in three distinct AI/AN communities that varied widely in their geographies, cultures, histories, and research capacities. The process of forming such a partnership was an experiment in how to effectively communicate and mobilize our individual strengths to achieve a point of mutual coordination.

### Survey Development

A goal of CEIGR was to conduct public deliberations in three tribal communities on genetic research (Hiratsuka et al., 2019, see also Hiratsuka et al., 2020a and Reedy et al., 2020b). To achieve this goal, it was necessary to engage in a multi-year process of building relationships between the University staff and community sites, working towards a consensus on the appropriateness of deliberation as a form of engagement in each respective community, and developing an approach to how this work could be conducted with a common goal.

To gain awareness of the variety of potential engagement practices, the consortium conducted a scoping review that summarized current practices regarding participatory research conducted with AI/AN communities (Beans et al., 2019; Woodbury et al., 2019a). An ancillary objective of the scoping review was to find out if deliberation had been done in tribal communities and, if so, to determine whether this method was an acceptable form of engagement in the tribal settings. We found that much of the research on deliberation that addressed minority group members focused on ensuring minority populations participated in deliberative forums (O’Doherty & Burgess, 2009; see also Ratner, 2004, Carson et al., 2013) or on conducting ‘enclave’ deliberation among minority populations in order to inform a larger deliberative process (Karpowitz et al., 2009). Our coordinated effort to design and implement a cross-site approach to deliberation exclusively in AI/AN tribal settings had no precedent and led to deepened relationships among CEIGR partners, putting into practice CEIGR’s partner-driven research approach.

In considering materials that might be shared with individuals participating in each site’s public deliberation, the site leads perceived that the AI/AN community members in their respective locations would have differing understandings, attitudes, beliefs, and preferences based on their different exposures to genetic research. After extensive discussion on how to meet these unique informational needs, one site lead suggested conducting a survey to determine community member interests and educational needs and using the resulting dataset to develop targeted briefing materials and expert presentations for the public deliberations.

After other site leads agreed to the approach, the Center’s students, community-based researchers, and faculty members brainstormed potential survey topics, including cultural cognition of scientific consensus; Indigenous spirituality; ancestry and migration; data sovereignty; attitudes toward research in general; knowledge of genetics; and, attitudes and beliefs related to biorepositories, precision medicine, and genetic testing. As a group, Center members refined the survey purpose and list of survey topics; in smaller workgroups, they developed and nominated existing survey items or scales to be considered for inclusion in the survey. This process resulted in a composite 137-item pilot survey using 4 scales previously tested in other populations as well as four scales we designed specifically to address AI/AN community specific concerns, such as specimen handling.

As there was disagreement on the topics for inclusion and because the site leads disagreed on the tone and wording of the items, the AI/AN site leads and OU PI decided to employ cognitive interviewing as a means of incorporating AI/AN community member viewpoints into the survey development process. Cognitive interviewing is a process in which draft survey questions are administered while collecting additional verbal information about the survey responses, which is used to evaluate the quality of the response or to help determine whether the question is generating the information that the author intends (Willis, 2010). Through the cognitive interviewing process, the CEIGR partners found that AI/AN participants at all sites understood the instruction text, and items and scales generated no cognitive difficulties. However, participant responses indicated a need for wording changes in survey instructions and items to improve understanding of key constructs. Problems noted included participants being unfamiliar with some terms used describing genetic and biological specimens. In several cases, participants’ written response in the survey and verbal response in the interview did not align. In several cases, written response in paper survey and verbal response in the interview did not align. In one of the CEIGR partner sites, when participants did not know what a word or phrase meant, they would mark down a neutral answer for the most part in one community however in another community when participants didn’t understand a word or phrase they would often mark down strongly disagree. These differences in response item selection across sites highlighted local item response behavior that could lead to response misinterpretation at a site level and when responses are aggregated across sites.

Cognitive interview results per item were reviewed by the CEIGR partners to select items and determine item rewording that would accommodate and correct for potential sources of response error, issues with item interpretation, and face validity. Following a shared cognitive interviewing process used across the three sites and led by the CEIGR team, a 52-item survey was finalized. The final survey included items that addressed several topics including conduct of research; personal beliefs; perceptions of researchers and research regulations; benefits and harms of research; research oversight; genetic testing benefits and risks; direct-to-consumer testing; and, demographics for use in AI/AN communities. The cognitive interviewing process took longer than the site leads had initially intended, as training of site staff was needed and because one site had delayed recruitment. Fortunately, in the planning of the deliberations, it was decided that briefing materials were not needed, and the site leads moved forward with fielding the cross-site survey at their sites. The cognitive interviews and cross-site surveys allowed each site to engage and better understand their communities’ reactions to delicate topics ahead of the upcoming deliberations.

### Deliberation

As mentioned above, a goal of the Center was always to conduct public deliberations on genetic research across all of our partner sites. The scholarship on public deliberation suggests it is a particularly promising approach for promoting deeper discussions around complex issues like genetics research and advancing public deliberation in AI/AN communities has been and continues to be a major focus of the Center (Hiratsuka et al., 2020a; see also Reedy et al., 2020b). There was an understanding that the Center’s effort to facilitate these deliberations was happening across diverse tribal settings, each with their own goals for public deliberation. The challenge was figuring out how to design the deliberations to have common research components and to remain sensitive to the unique needs and priorities of each partner site.

In July 2018, after months of conference call discussions that seemed to render the task of cross-site deliberations unattainable, representatives from each site held an in-person meeting to discuss the feasibility of designing three public deliberations that addressed distinct goals while somehow maintaining the integrity of the project as a cross-site initiative. Building consensus across all partner sites, while also preserving the preferences unique to each community setting, set the stage for discernable levels of tension and disagreement, but hindsight underscores these tensions and the process of navigating them were a critical part of the planning phase. This particular meeting represented a number of important firsts for our consortium: it was the first opportunity for all sites to work directly with the deliberation scholar selected to facilitate all of our deliberations; it was the first meeting held at an otherwise “neutral” location not affiliated with any of the partner sites; and, it was the first of many coordinated efforts where team members other than the site leads were making critical decisions about the direction of the Center’s work. These *firsts* introduced an entirely new set of interpersonal and cross-site dynamics to the research planning process; it is possible that what might have been perceived as challenges at that time would have been difficult to work through without the collective commitment to work towards something larger.

Face-to-face meetings had always been a central feature of our consortium, in part because of the geographically dispersed locations of the partner sites but also because these in-person meetings provided the opportunity to build trust and interpersonal relationships across sites. That particular moment in the planning process also underscored the importance of the in-person meeting format for moving the work of our unique Center along; the time and space needed for all partners to adequately express their perspectives and establish a sense of collective goals required the opportunity to workshop ideas that conference calls and emails could not. Balancing the local and collective goals in the deliberation planning was a lofty task and possibly the first extended test of collaboration we had embarked upon.

To date, we have completed deliberations at each of our three community partner sites (Hiratsuka et al. 2020a, Reedy et al., 2020b). The truly unique feature of our deliberations, aside from being conducted in exclusively tribal contexts with AI/AN participants, is that they were designed around the questions, priorities, and social dynamics associated with each community site. For instance, SCF had been involved in pharmacogenetic research for a number of years (Hiratsuka et al., 2020b), with several research projects exploring AI/AN views on biological specimen use (Hiratsuka et al., 2012a, Hiratsuka et al., 2012b, Dirks et al., 2019) and preferences for the conduct of pharmacogenetic research (Avey et al., 2016; Beans et al., 2020; Shaw et al., 2013). The questions of interest for this site were therefore focused on community preferences for return of results from genomic research (Hiratsuka et al., 2020a) as the site was conducting pharmacogenetic research projects and was seeking to improve understanding of community preferences for dissemination of findings. The CN, on the other hand, was beginning to think about the role of genetic research for its own community. While MB had participated in select genetic studies over the years (Claw et al., 2020; Khan et al., 2018; Fohner et al., 2015; Woodahl et al., 2014), many questions remain about perceptions of genetic research that had never been asked of its community before the deliberation. Findings from this deliberation have been disseminated to the appropriate tribal authorities for review and consideration (Reedy et al., 2020b). Finally, the deliberation at MB posed very different questions than the two previous sites, in part because this site is not integrated into a specific tribal health care delivery system with defined research policies and processes, but is a private AI-owned research entity working toward the development of a biorepository within a tribal jurisdiction. These particular circumstances prompt important questions to ask of community members, especially about developing solutions for expanding research capacity and how to govern genomic data in ways that honor tribal sovereignty. These distinctions across sites necessitated different deliberation questions, and our approach reinforces the prospect of designing research that is both applicable across diverse sites and also directly responsive to the goals and needs and local communities.

Deliberations can yield informed and egalitarian discussions and are particularly valued by members of some minority groups (Gastil et al., 2010; Goold et al., 2005; Knobloch et al., 2013; Wang et al., 2015), but until now there has been little work that examines public deliberation exclusively in Indigenous contexts (Carson et al., 2013, Reedy et al., 2020a). Our work in deliberation became a feasible approach for engagement and dialogue in AI/AN contexts only through a coordinated process of melding disciplinary expertise with mutual learning and cooperation across all sites.

Our cross-site research initiatives all strive to strike a balance between collective goals and local priorities. It is never guaranteed that all of our research initiatives will resonate across the three sites, nor will they necessarily lead to generalizable results, but the process of working together and creating spaces for different team members to lead as necessary ensures that we are constantly learning and challenging each other to explore more ethical approaches to genetic research. Face-to-face meetings were key to facilitating the process of developing the surveys and the deliberations described above; in both cases, it was only after we transitioned from conference calls and emails to in-person workshop sessions that substantial progress began.

In the site-specific planning sessions, team members co-developed the deliberation facilitator guide to be used at the site which laid out individual responsibilities of team members, processes for deliberative activities, and the specific materials and questions that would be asked to site deliberation participants. It became clear early in the process that the planning work being done at one site, regardless of specific deliberation questions, could be of use to the other sites. As such, sharing of document templates and planning experiences across sites occurred regularly. Another specific example of sites engaging one another is the use of case scenarios at each site. Case scenarios are hypothetical depictions centered around topics related to each deliberation, designed to be read by deliberants and to facilitate discussion that includes reactions and considerations of how each scenario resonates within each community. While not initially part of the collective approach to deliberations, the success of the case scenarios at the first site’s deliberation prompted their continued use at the other two sites. The scenarios enabled participants to consider issues related to genomics research in very personalized- albeit hypothetical- ways. The scenarios were co-designed by the sites and the CEIGR researchers and were tailored in ways that drew upon local concerns, terminology, family and kin structures, current events, and specific tribal experiences.

The specific approaches outlined here offer tangible considerations for how other research partnerships might approach the co-design and implementation of research in ways that align with one another and maintain the integrity of local community goals. The description of our sequential use of cognitive interviews, surveys, and deliberation provides a blueprint for how to implement research across unique sites in ways that is reflective and builds upon the lessons at each site. We have also learned that the interpersonal nature of partnership building requires considerable attention and that our ability to conduct ethical and engaged community research must begin with our willingness to engage each other and appreciate the differences across sites.

## Power Distribution

CEIGR is unique in that the University site is not a coordinating center; rather, each partner- SCF, the CN, and MB- shares responsibility in the development of all aspects of the Center from administrative functions, the development of research agendas, and manuscript development. This commitment to power sharing and effort distribution is evident in the budget allocation. Over half of the CEIGR’s direct costs are equitably distributed to the community partners so that each site can conduct site-specific work (e.g., data collection) without fiscal stress and in a manner that contributes to the collective goals of the Center. This budget structure reflects the centrality of the partner sites in accomplishing the kind of work prioritized at each site and it contributes to the collective effort to accomplish activities in ways that are appropriate at each site. Our Center recognizes this as a more inclusive partnership model that elevates community-based investigators and is more responsive to community-driven initiatives, thereby establishing a more equitable approach to ELSI work in AI/AN contexts.

### Decentralizing Budgets

Academic and community standards and expectations related to the research process are often misaligned. Budget decentralization creates opportunities to prioritize activities and personnel outside of conventional academic achievements and faculty. The equitable distribution of funds across all partner sites also promotes a plasticity of leadership within CEIGR that presents opportunities for partner sites to lead specific initiatives. The distribution of funds across sites presented an opportunity for sites to manage budgets according to their own research agendas, but it also underscored differences in each site’s experience creating and managing large National Institutes of Health-funded research budgets. The SCF site lead, for example, developed a budget that was used as a template for the other two sites, thereby helping to build capacity in the other sites, and to assist in the coordination of CEIGR activities.

One specific goal of our Center is the training and advancement of AI/AN junior scholars and early stage investigators. To this end, the University budget maintains designated support for undergraduate and graduate students and post-doctoral researchers. Consistent financial support is an ideal that many students cannot always achieve, yet it is necessary for realizing the increased representation of AI/AN scholars in academia. Through consistent support from CEIGR, AI/AN students are able to be a part of the collective efforts of the Center while simultaneously building their own research networks for their future. Further, students benefit from participation on many CEIGR projects and contribute in leadership roles alongside all of the Center’s members. The various disciplines and professional levels represented in the consortium have created a malleable space that allows for growth at all professional levels.

### Manuscript Writing and Dissemination

Following the CEIGR practice of open, transparent group conversation on research design, presentations and manuscripts describing CEIGR work have developed in a similar manner. Development of a process to co-develop manuscript ideas, coordinate writing, and mentor partners on the publishing process were key steps in implementing a process for manuscript development.

Our processes to invite writing contributions ranged from a process to share manuscript proposals across the partnership to a bi-weekly manuscript workshop session. We sought to involve all members in consensus-building processes and writing teams. To advance manuscript development, three in-person meetings have focused solely on manuscript concept development with facilitated conversations. We implemented virtual writing sessions whereby all consortium members participate via conference call. Finally, using a tracking spreadsheet developed by the SCF, the Center has developed a tracking and consortium authorship concept.

Of note within the partnership, a large number of non-academic partners are actively involved in manuscript authorship. Just as publishing is necessary for those in faculty positions/settings, so is it vital for those partners actively pursuing grant funding. Graduate students are mentored and actively participate in the manuscript writing process, participate as part of larger writing teams and lead the conceptual development of specific manuscripts. Further, all CEIGR partners have participated in the dissemination of our work in a variety of formats, including poster presentations, oral presentations, round table discussions, community presentations and townhalls, professional panels, radio shows, webinars, and other community-specific outlets.

Our Center works across multiple tribal settings, and all partners coordinate their independent efforts to navigate the tribal research review processes our work must undergo. Akin to data ownership in tribal settings (Hudson et al., 2020; Woodbury et al., 2019b), manuscripts describing processes and outcomes associated with tribes, tribal data, and tribal members can be subject to tribal oversight (Blue Bird Jernigan et al., 2015; Hiratsuka et al., 2017). Tribal entities may not wish to have their research results published in journals or disseminated in certain public spaces (Tsosie et al., 2019). Navigating the internal manuscript and abstract review and approval processes for each tribal partner requires forethought and planning to orchestrate timely approvals prior to dissemination activities. CEIGR partners have dual roles of staffing the tribal processes and being subject to the processes.

Our Center operates according to an informal principle of “coordination without a single coordinator” and we understand that achieving collective goals requires respectful collaboration, ongoing communication, and fluid leadership that responds to the emergent needs and challenges inherent in doing community-engaged research. This model of partnership alleviates the potential for any one site to be over-burdened and creates opportunities for each site to contribute expertise and assume leadership roles.

## Co-learning/Capacity building

The Center for the Ethics of Indigenous Genomic Research, as introduced earlier, is a multidisciplinary consortium comprised of researchers with expertise in genomic sciences, anthropology, public health, communication, political science, bioethics, Native American studies, and a diversity of lived experiences to inform our approach to research and engagement. Beyond the assortment of disciplinary backgrounds within our Center, differences in the capacity and experience of our partners was also key to moving CEIGR’s goal of cross-site research activities in all partner sites. As one partner in our Center had extensive expertise in conducting original research in their own site, other partners were looking to grow their experience beyond data collection to research design. Discrepancies between partners presented discernable opportunities to work together in ways that promoted capacity building through the execution of cross-site research activities. Data collection needs presented opportunities to co-learn new methods, which in turn created opportunities to explore new approaches to data analysis and sharing. There was an iterative, building-block nature to our collective approach to the research process, so that our ability to complete cross-site research activities as a Center rested on our collective skills and willingness to learn from one another. Co-learning was an essential piece of our research process; the ebb and flow of mutual learning opened up space to acknowledge our needs and enable everyone to contribute. The process of conducting cognitive interviews and deliberations at all three partner sites underscores the importance of capacity building through co-learning.

### Cognitive Interviews

This community-site driven process strengthened synergy across the consortium, built community site capacity and informed future empiric recruitment and data collection. Within the development and implementation of a cross-site survey as describe in detail above, the SCF site led the overarching scientific approach. To develop and implement consistent data collection processes, a community partner training was conducted, and support of community partners occurred. SCF led this initiative by first facilitating dialogue across the consortium during in-person meetings in April 2017 and August 2017 and between in-person meetings via teleconference and email. Consortium members put forward survey items covering a variety of topics including: direct-to-consumer testing, genetic testing risks and benefits, science and society, and personal beliefs about biological specimens. We used cognitive testing across three sites to systematically evaluate the appropriateness of the survey questions.

SCF developed the cognitive interviewing plan. Staff from the CN and MB traveled to Anchorage, Alaska where SCF hosted a cognitive interview training workshop that included: hands-on practice on recruitment, informed consent, survey data collection, data entry, interviewing, and interview notes. All three sites were trained using the same interview questions, survey items, data collection tools, and data entry Excel sheets that were used during data collection. Once cognitive interview data collection at each site was complete, the sites discussed the findings via a video conference call. Cognitive interview findings were discussed as well as implications of those findings on the survey items. From this discussion the cross-site survey was developed with site specific items.

Through the cognitive interview process, staff members at each site were able to gain confidence in research skills. Staff learned to comfortably discuss study aims and answer questions about the study, gained familiarity with recruitment locations, and were able to practice systematic and interactive approaches involved in the conduct of research. The confidence and familiarity with conducting research prepared the community sites for survey and deliberation work.

### Deliberation

The deliberation planning process was, as mentioned above, a test of our collective commitment to develop and implement cross-site work. Community and University partners met at a face-to-face meeting to come to consensus on the cross-site approach we were going to take as a Center. Together, we decided that the process of each deliberation would be the same, but the content discussed would be site-specific. To accomplish this task a core deliberation team, comprised of individuals from the University of Oklahoma (OU) and the University of Washington (UW), worked with each partner site to design the specific deliberation details. Each planning group maintained their own series of conference calls, complemented by a set of regularly scheduled consortium-wide calls. Maintaining a separate set of conference calls for each site and for the entire Center could be cumbersome and time consuming, but was also necessary for allowing each site to pursue their own directions independent of group consensus.

This model of cross-site deliberation planning revealed some unexpected group dynamics. SCF, for example, provided tremendous leadership early in the development of documents needed for the deliberation protocols. Their willingness to share these documents provided critical assistance to the other sites as they began their deliberation planning. CN was the first site to conduct their deliberation. As a result of being the first site in our consortium to conduct a deliberation, their insight and experience proved crucial in helping the other sites finalize their deliberation plans. MB was the final partner to conduct their deliberation, and the methods that we had similarly employed at each site were received quite differently at this community site; this preliminary finding suggests that the MB deliberation team may offer some important feedback on the evaluation of our deliberative approach because it was received differently at their site. The cross-site deliberations presented each site an opportunity to lead, reinforcing the importance of conducting research in a way that promotes equitable opportunities to lead, mutual learning among all partners, and capacity building. The deliberations centered around local questions stemming from specific needs and concerns of each community; as such, the deliberations provided concrete input on issues of central importance to each community. A preliminary report summarizing input from each deliberation was sent to all deliberants and revised according to their recommendations, before final reports were disseminated to the appropriate tribal leaders and administrators at each site for consideration of next steps. We will be reporting on those next steps as they are decided upon locally.

## Discussion: Overcoming Challenges

### Navigating Capacity

Building Center and individual community site capacity has required much patience and persistence of each CEIGR member but it was necessary to accomplish equitable cross-site work. Together, we have learned to allow one another to take the lead and allow the group with expertise to lead when necessary. In some cases this had been University staff, in other cases it has been community site staff; often, we lead together. The deliberations, as discussed above, were an example of the University and community sites needing to work together and recognizing the need for expertise on the deliberation. The invitation of the UW deliberation expert created an equal playing field for mutual learning of all CEIGR members and facilitation by the deliberation expert opened up space for all consortium members to bring specific strengths forward to conduct the deliberations. While the introduction of new team members and new approaches requires careful attention to the effect on the group dynamic and research direction, the absence of a strict hierarchical structure within CEIGR promotes an environment that is receptive to new partners and new ideas.

### Building Trust through Communication

As noted, maintaining face-to-face communication in constant and consistent ways is key, as the dynamics of the consortium are always changing. There is a tremendous amount of interpersonal communication necessary to ensure that we achieve enough common footing across the consortium to allow for equitable research across the consortium. Further, regular meetings provide opportunities to establish agreement on broad, ethical principles thereby strengthening the foundation upon which diverse stakeholders can manage power dynamics and building relationships (Hoover et al., 2019). Achieving this common footing requires continual check-ins with each other to understand the changing needs of each partner. The differential capacities of each site mean that one site may experience feelings of “being territorial” over certain research activities or that one site may struggle to garner recognition in comparison to the successes of other sites. One strategy for overcoming this is to institute face-to-face meetings as a regular activity of the center. It is essential for all partners to be able to communicate changing comfort levels with center activities and regular meetings that all partners expect and plan for create a space for being able to communicate feelings about things.

Building trust in AI/AN communities is the guiding imperative in the work we seek to do. Centering research around community-placed researchers and community-based organizations—as opposed to academic institutions removed from the communities most impacted by research—establishes a more consistent presence and places the entire research process within the socio-political, historical and cultural contexts that shape the experiences of community members. The prolonged presence of community-placed researchers helps establish long-term relationships, facilitates trust, and provides opportunities to receive community input and to incorporate community feedback to improve data collection strategies and the questions we ask.

### The Value of ELSI Work

The CEIGR consortium is comprised of several organizations with complementary, but non-identical research priorities and capacities that differentially affect their interest in and ability to support a range of ELSI research projects. Research priorities affect the perceived value of ELSI because conducting this research imposes opportunity costs on CEIGR partners. For example, conducting ELSI research consumes personnel, material, and financial resources that could be dedicated to other activities. Similarly, time and resources spent developing expertise in ELSI shapes future research opportunities, since funding decisions are based in part on prior research experience. The perceived value of ELSI is also affected by differences in research capacity that alter the nature and scope of commitments that CEIGR partners must make in order to contribute to the consortium’s research projects. Partners that must first build capacity in order to conduct ELSI research will commit more resources to these projects than those that already possess most or all of the necessary expertise. The perceived value of ELSI will be greater among organizations with research priorities that are advanced by engaging in this area of research and that can obtain the benefits of ELSI without substantial investments in capacity.

In most contexts, the focus on normative questions within ELSI research means that its impacts on human health can be indirect and difficult to quantify, especially by comparison with those arising from basic science, translational, and clinical research (Parker et al., 2019). Given the sensitive nature of genomics as the subject matter, a history of research exploitation, and lawsuits and policy change in tribal contexts, ELSI work seems essential to ameliorating these issues for the increased benefit and quicker implementation of state-of-the-art research (Walker & Morrissey, 2012). The deliberative work completed by CEIGR, for example, is based on systematic inquiry into ELSI issues but the actionable direction and policy guidance that emerged from this work is a direct service to the partnering AI/AN communities. In addition, members of the CEIGR consortium have advocated for enhanced protections for research participants and increased commitment to community engagement in and shared control over research processes (Beans et al., 2019; Chadwick et al., 2014, 2019; Hudson et al., 2020; Tsosie et al., 2019; Woodbury et al., 2019b). These actions offer ethical and scientific benefits, but can also increase the cost, complexity, and duration of research (Buffalo et al., 2019). Funding mechanisms that support inquiries into ELSI research are essential for many AI/AN communities working to give voice to concerns that have been unheard or underrepresented in conventional research arenas, and to elevate the urgency of these concerns to be on par with larger, more powerful institutions.

Despite such challenges, there is evidence of sustained interest in the findings and recommendations of ELSI research. In particular, the kind of robust community engagement in health-related research studied and advocated by CEIGR partners has received attention from researchers and communities interested in utilizing these approaches in their own research. CEIGR takes seriously the significance of sharing our successes and challenges as it relates to addressing persistent questions in ELSI and to cultivating a new relevance for ELSI work for tribal and other extra-jurisdictional communities, for whom research protections have often been more reflective of colonial constructs & agendas (Hudson et al., 2020), rather than in service to the unique political designations and worldviews of sovereign AI/AN tribes and other Indigenous peoples.

## Conclusion

Our Center emerged in response to the persistent, unresolved issues that kept all too many tribal nations from participating in genomic research. We coupled this understanding with a commitment to pursue engagement in ways that promoted increased representation, dialogue, and inclusion of AI/AN researchers and community perspectives. The partnership itself is always in a constant state of becoming. Moving forward, we continue to be guided by these principles and have structured the direction of CEIGR in ways that shift power away from a traditional university-PI model toward a model of engagement and research that is inclusive of community-based investigators and prioritizes community-driven inquiries.

At the center of our collective efforts is respect for tribal authority and research oversight in all aspects of this work. There is no doubt that histories of missteps with respect to tribal authority have done significant damage to research progress in AI/AN communities and our respect for these processes, both in gaining approval for specific projects and also in the review and approval of manuscripts, has resulted in a kind of research process that challenges conventional timelines and outputs. Nonetheless, in our fourth year as a Center, we have achieved some significant milestones allowing for cross-site efforts led by the tribally based partners. We funded a series of local pilot projects that permitted each of our community partners to articulate a research agenda that could be brought into dialogue with us and with the other partners. We jointly developed and administered the first systematic survey of attitudes toward genomics in AI/AN communities ever attempted. We developed deliberations in each of our community partners. Finally, we had successes in areas of professional advancement, supplemental funding, and program development.

CEIGR was founded upon a commitment to do the kind of work that can be difficult to fund under many established funding mechanisms—the building of relationships with tribal communities—in our case to advance honest dialogue about the place of genomics in AI/AN communities. This commitment rested upon the collaboration between three community partners, new to each other, to jointly articulate a research agenda. This work cannot be the standard kind of hypothesis testing that has shaped research for so long. Rather, this work requires an openness to community concerns and adherence to a research agenda that can be difficult to specify and negotiate between multiple partners. Partnership building is not without challenges, but a mutual commitment to community-centered research and a concerted effort to open communication is key to finding success within this model.
